# The Role of Brain Plasticity in Neuromuscular Disorders: Current Knowledge and Future Prospects

**DOI:** 10.3390/brainsci14100971

**Published:** 2024-09-26

**Authors:** Paolo Alonge, Giulio Gadaleta, Guido Urbano, Antonino Lupica, Vincenzo Di Stefano, Filippo Brighina, Angelo Torrente

**Affiliations:** 1 Department of Biomedicine, Neuroscience and Advanced Diagnostics (Bi.N.D.), University of Palermo, 90127 Palermo, Italy; paolo.alonge01@unipa.it (P.A.); antoninolupica@aopapardo.it (A.L.); vincenzo19689@gmail.com (V.D.S.); angelo.torrente@unipa.it (A.T.); 2 Neuromuscular Unit, Department of Neuroscience “Rita Levi Montalcini”, University of Turin, 10126 Turin, Italy; giulio.gadaleta@unito.it (G.G.); guido.urbano@unito.it (G.U.); 3 U.O.C. Neurologia, Azienda Ospedaliera Papardo, 98121 Messina, Italy

**Keywords:** brain plasticity, neuromuscular disorders, non-invasive brain stimulation, transcranial magnetic stimulation, transcranial direct current stimulation, clinical neurophysiology, rehabilitation, muscle dystrophy, inflammatory myopathy, spinal muscular atrophy

## Abstract

**Background/Objectives**: Increasing evidence shows an involvement of brain plasticity mechanisms in both motor and central manifestations of neuromuscular disorders (NMDs). These mechanisms could be specifically addressed with neuromodulation or rehabilitation protocols. The aim of this scoping review is to summarise the evidence on plasticity mechanisms’ involvement in NMDs to encourage future research. **Methods**: A scoping review was conducted searching the PubMed and Scopus electronic databases. We selected papers addressing brain plasticity and central nervous system (CNS) studies through non-invasive brain stimulation techniques in myopathies, muscular dystrophies, myositis and spinal muscular atrophy. **Results**: A total of 49 papers were selected for full-text examination. Regardless of the variety of pathogenetic and clinical characteristics of NMDs, studies show widespread changes in intracortical inhibition mechanisms, as well as disruptions in glutamatergic and GABAergic transmission, resulting in altered brain plasticity. Therapeutic interventions with neurostimulation techniques, despite being conducted only anecdotally or on small samples, show promising results; **Conclusions**: despite challenges posed by the rarity and heterogeneity of NMDs, recent evidence suggests that synaptic plasticity may play a role in the pathogenesis of various muscular diseases, affecting not only central symptoms but also strength and fatigue. Key questions remain unanswered about the role of plasticity and its potential as a therapeutic target. As disease-modifying therapies advance, understanding CNS involvement in NMDs could lead to more tailored treatments.

## 1. Introduction

Primary neuromuscular disorders (NMDs) include a broad group of diseases whose clinical mainstays are progressive weakness, reduction in muscular trophism, and exercise intolerance although the manifestations still may be various. Whilst the purely neuromuscular component has been widely explored through biomarkers of peripheral damage [1], central nervous system (CNS) alterations are frequently observed, but scarcely characterised [2]. An emblematic example is represented by the cognitive deficits in Duchenne muscular dystrophy, where dystrophin is well-known to be involved in brain metabolism, although the mechanisms underlying its role in neuronal plasticity have still only been partially investigated [3].

Brain plasticity is a process that involves multiple mechanisms owned by the CNS leading to changes to adapt to internal or external stimuli. Plasticity is a natural CNS property regarding physiological or pathological conditions that involve both structural and functional processes [4]. Moreover, neuronal plasticity may be observed in the short term (i.e., milliseconds to minutes), for example, to respond to sensory stimuli, or in the long term (i.e., hours to days), such as for behavioural changes due to experiences [5]. Long-term plasticity is dependent on mechanisms of neuronal firing or circuitry called long-term potentiation (LTP) and long-term depression (LTD). 

It is possible to safely study some aspects of the cortex such as excitability using non-invasive brain stimulation (NIBS) techniques. One of these means is transcranial magnetic stimulation (TMS), which is based on Faraday’s electromagnetic induction principle: a brief high-intensity electric current (i.e., TMS pulse) travelling through a coil induces a magnetic field that passes through the skull and can induce a second current over the cortex [6]. The most used TMS mean to study cortical excitability is to stimulate the primary motor cortex (M1), recording the motor evoked potential (MEP) from the contralateral abductor digiti minimi (ADM) or abductor pollicis brevis (ABP) muscle. TMS plays an important role in the evaluation of brain plasticity since it allows for the assessment of cortical properties during different states or after induced conditioning [7]. Some protocols include a single-pulse TMS (spTMS), while others use a paired-pulse TMS (ppTMS) to evaluate mechanisms of intracortical inhibition (ICI) or facilitation (ICF) based on the interstimulus interval (ISI). Furthermore, repeated TMS (rTMS) with high-frequency (≥5 Hz) pulses induces facilitatory effects on cortical excitability, while low-frequency (≤1 Hz) rTMS shows inhibitory effects, respectively [8]. Similar and even more pronounced modifications can be induced with special rTMS protocols, such as theta burst stimulation (TBS). Typically, here 50 Hz bursts of three pulses are delivered with a frequency of 5 Hz with a continuous (cTBS) or intermittent (iTBS) pattern producing inhibitory or excitatory effects [9]. Another safe and easy-to-use NIBS technique able to induce brain plasticity changes is transcranial direct current stimulation (tDCS). This tool modulates cortical excitability through the administration of small anodal or cathodal currents on the scalp, with facilitatory or inhibitory effects, respectively [10].

The connection between CNS and the periphery (i.e., sensory afferents, muscle and peripheral nervous system) has been traditionally conceived as rigid, while recent evidence suggests the presence of plastic mutual influence. For example, animal studies demonstrated how fast-type motoneuron (MN) properties can be modified by chronic muscle overload in rats [11]. Regarding brain plasticity mechanisms in patients affected by muscle dystrophy, a reduction in ICI has been observed and referred to as a compensatory mechanism to obtain as much muscle strength as possible [12]. Patients’ brain functional reorganisation can be investigated by functional magnetic resonance imaging (fMRI) during a hand movement task. For instance, patients affected by pure motor neuropathy showed increased activation of the contralateral primary motor cortex, ipsilateral primary motor cortex, and supplementary motor area compared to healthy subjects or patients with pure sensory neuropathy [13]. Finally, preclinical studies demonstrated how repeated magnetic stimulation (rMS) can induce LTP effects and enhance the expression of brain-derived neurotrophic factor (BDNF), possibly representing a therapeutic strategy [14].

Currently, most studies in the field of NMDs focus on muscular features and rehabilitative approaches. However, there is a lack of understanding of the potential involvement of the CNS in these disorders, alongside central compensatory mechanisms. Evaluating brain and neuronal changes in terms of neuroplasticity could enhance our knowledge of the pathophysiology of NMDs and broaden therapeutic opportunities.

The purpose of the present narrative review is to summarise the current literature regarding brain plasticity in NMDs. This approach may give new research inputs, in the perspective of future interventional studies that may exploit plasticity mechanisms to improve rehabilitation programs and ameliorate patients’ quality of life. 

## 2. Materials and Methods

The inclusion criteria for the research were based on the PCC (population, concept, context) criteria listed below:

Population: we included articles which investigated brain plasticity in NMDs, including muscular dystrophy, myopathies, myositis, and spinal muscular atrophy (SMA), both on patients and preclinical models. We did not include studies on amyotrophic lateral sclerosis since it is already well consolidated in the literature that the disease presents brain changes due to the first motor neuron involvement. The authors also chose to exclude myasthenia gravis from the selection to focus on disorders with predominant or structural primary muscle involvement.

Concept: the research goal was to review the current evidence on brain plasticity, with a specific focus on neurophysiologic measures assessed through TMS, tDCS, or fMRI.

Context: no specific limits regarding age, sex, cultural and geographic differences were considered.

The literature research has been conducted using PubMed and Scopus databases, looking for English full-text original articles (including case reports and case series) from year 2000 onwards about brain plasticity in primary NMDs. As exclusion criteria for the selection, we set non-original articles (e.g., literature reviews or editorials), articles not written in English, and publication before year 2000. The search enquiry used was the following: (“transcranial magnetic stimulation” OR “tDCS” OR “TMS” OR “Transcranial Direct Current Stimulation” OR “fMRI” OR “functional magnetic resonance” OR “brain plasticity” OR “neuronal plasticity” OR “SNC”) AND (“neuromuscular disease” OR “muscular dystrophy” OR “motor neuron disease” OR “myopathy” OR “myositis” OR “muscle dystrophy” OR “spinal muscular atrophy” OR “dystrophin” OR “smn1” OR “dystrophy”) NOT review NOT stroke NOT amyotrophic lateral sclerosis NOT dementia. Four authors (AT, GG, GU, PA) examined the outputs of the research, considering the results since year 2000 until the time of the search (16 July 2024). First, the titles were checked (excluding double matches, off-topic or non-English papers), and then the remaining abstracts were examined to judge whether the articles suited the topic of the review. For each step, the authors were asked to express a judgement on whether to include (Y) or not (N) in the article. If there was a majority of Y or a parity between Y and N, the article was included in the following reviewing step. Finally, the resulting full-text papers have been examined by the authors and reviewed here. In addition to the search output, authors were allowed to look for more articles using a free search line on the databases based on their knowledge.

## 3. Results

The search provided an output of 2206 results (605 and 1601 from PubMed and Scopus databases, respectively). Overall, there were 178 duplicates and 2 articles not written in English (1 in Chinese and 1 in Portuguese language). So, 2026 titles had been examined for topic suitability, leading to 67 potential articles of interest. Among the 67 abstracts, 49 were selected for full-text examination. During the last phase, 3 articles were excluded because there was no full text available, for a final output of 46 papers. Based on the topic addressed and on the number of results, articles were divided into three main groups: dystrophinopathies, myotonic dystrophy type 1 (DM1) and other myopathies. After a full-text examination, 13 off-topic papers were excluded. The results are summarised in Table 1 (dystrophinopathies), Table 2 (DM1) and Table 3 (other myopathies).

### 3.1. Dystrophinopathies

Dystrophinopathies are genetic disorders linked to pathogenic variants in the *DMD* gene, which encodes the dystrophin protein. Dystrophin plays a crucial role in maintaining the structural integrity of muscle cells, but it also has significant functions in the CNS. The brain expresses different isoforms of dystrophin, including the full-length Dp427, which is found in selected CNS regions such as the hippocampus, cortex (including pyramidal neurons), amygdala, and cerebellum, both in Purkinje cells and cerebellar nuclei [15]. Additionally, shorter isoforms like Dp140 and Dp71 are expressed in the brain, with Dp71 playing a key role in glutamatergic synaptic transmission and hippocampal synaptic plasticity [16,17]. Altogether, these isoforms are essential for normal cognitive function, and their deficiency is associated with various degrees of central impairment in DMD patients.

#### 3.1.1. Dp71 Deficiency and Its Impact on Synaptic Plasticity

Dp71, a shorter isoform of dystrophin, is highly expressed in the brain, mainly in glial cells, where it contributes to the clustering of potassium channels (Kir4.1) and aquaporin channels (AQP4) in perivascular domains [18]. This localisation is crucial for maintaining the homeostasis of the extracellular environment, particularly in relation to ion regulation and water transport, which has downstream effects on neuronal function, including in the cerebellum [16,19]. The loss of Dp71 has further consequences for synaptic transmission and plasticity, particularly in the hippocampus. In Dp71-null mice, there is an abnormally increased basal neurotransmission in the CA1 hippocampal region, accompanied by reduced paired-pulse facilitation (PPF) and LTP [16,20,21]. These alterations are linked to changes in glutamatergic transmission at both pre- and postsynaptic levels, including disrupted clustering of glutamate receptors and altered presynaptic vesicular glutamate transporters [16,22]. Morphological studies in mice have shown that these alterations lead to a reduction in the readily releasable pool of synaptic vesicles, potentially impairing the replenishment process during LTP and thus compromising hippocampal synaptic plasticity [16]. Further investigations into Dp71-deficient mice have revealed that Dp71 contributes to the strength of glutamatergic synaptic transmission to Purkinje cells, particularly at synapses formed by climbing fibres (CF-PC) [23]. These changes lead to altered LTP, indicating that enhanced excitation and compromised synaptic plasticity are generalised disturbances affecting brain structures lacking Dp71 [19,23]. On the other hand, the absence of Dp71 also impacts inhibitory neurotransmission, specifically in the cerebellum. Studies have shown that the loss of dystrophin severely reduces inhibitory synaptic input to cerebellar Purkinje cells (PCs), as evidenced by a decrease in the clustering of GABA_A_ receptors and a reduction in functional inhibitory synapses [24]. This deficit in inhibitory transmission from PCs to cerebellar nuclei (CbN) neurons, especially during high-frequency activity, leads to increased baseline firing rates and reduced evoked firing in CbN neurons [15]. This suggests that the cerebellar circuits in DMD may have a diminished ability to process information and generate appropriate downstream activity, which could contribute to the motor and cognitive deficits observed in DMD patients.

#### 3.1.2. Functional Studies with fMRI, MRS, and TMS in Dystrophinopathies

Functional imaging studies have revealed significant alterations in brain function in dystrophinopathies. For instance, fMRI studies using voxel-based degree centrality (DC) and seed-based functional connectivity (FC) analyses have shown that DMD patients exhibit a significant increase in DC in the left dorsolateral prefrontal cortex (DLPFC.L) and right dorsomedial prefrontal cortex (DMPFC.R), coupled with a decrease in DC in the right cerebellum posterior lobe (CPL.R) and right precentral/postcentral gyrus when compared to healthy controls [19,25]. These functional abnormalities suggest an overactivation of the default mode network and executive control network, and a suppression of the primary sensorimotor cortex and cerebellum-visual circuit. Additionally, DMD patients showed stronger FC between the CPL.R and bilateral lingual gyrus, pre/postcentral gyrus and insular cortex, and DMPFC.R and precuneus.R, with attenuated FC between DLPFC.L and insular cortex [19,25]. Furthermore, Magnetic Resonance Spectroscopy (MRS) studies have provided further evidence. Increased levels of choline-containing compounds have been detected in the cerebellum and hippocampus of both DMD patients and *mdx* mice, suggesting altered membrane turnover and cellular integrity [26,27]. Positron Emission Tomography (PET) investigations have demonstrated altered glucose metabolism in the cerebellum and right sensorimotor cortex, possibly correlating with impaired neuronal function in these regions [28]. Transcranial magnetic stimulation (TMS) studies have produced conflicting results. For instance, one study found that dystrophinopathic patients had a higher motor threshold (MT) for magnetic stimulation compared to electrical stimulation, indicating reduced cortical excitability [29]. On the contrary, other TMS studies have reported no significant differences in cortical excitability between DMD patients and age-matched healthy controls [30].

**Table 1:** Summary of the key content from articles on dystrophinopathies.

Study	Aim of the Study	Main Findings	Type
Kreko-Pierce et al., 2022 [15]	To investigate potential dysfunction in the output of the cerebellum, downstream of Purkinje cell activity in juvenile wild-type and *mdx* mice.	Loss of dystrophin in pre-synaptic Purkinje cells reduces the dynamic range of synaptic transmission and firing in cerebellar nuclear neurons.	Preclinical
Daoud et al., 2009 [16]	To explore the role of Dp71 in neuronal function and identify mechanisms by which Dp71 loss may impair neuronal and cognitive functions in Dp71-null mice.	Loss of Dp71 causes altered synapse density in the adult brain, enhanced glutamatergic transmission and reduced synaptic plasticity in CA1 hippocampus. Dp71-null mice show selective behavioural disturbances and impairments in spatial learning and memory.	Preclinical
Lv et al., 2011 [17]	To investigate functional alterations of motor cortex connectivity with fMRI in DMD underaged patients.	Patients with DMD have lower grey matter concentration in the left primary sensorimotor cortex and decreased local synchronisation of spontaneous activity in the bilateral primary sensorimotor cortex and in supplementary motor area.	Clinical
Fort et al., 2008 [18]	To investigate the effect of Dp71 deletion on the membrane expression of the DAPs-Kir4.1 and -AQP4 complex in Müller glial cells.	Dp71 has a central role in the molecular scaffold responsible for anchoring AQP4 and Kir4.1 in Müller cell end-feet membranes.	Preclinical
Chaussenot et al., 2019 [19]	To identify changes in prefrontal cortex excitatory/inhibitory balance in a Dp71-null mouse model.	Deletion of Dp71 causes a shifting toward enhanced excitation in prefrontal cortex, alteration of AMPA-mediated transmission and reduced synaptic plasticity.	Preclinical
Miranda et al., 2011 [20]	To analyse the distribution of synaptic vesicles in CA1 hippocampal axospinous non-perforated-excitatory synapses of mice lacking Dp427 or Dp71.	The density of morphologically docked vesicles is increased and the vesicle size is reduced in mice lacking Dp427, while in Dp71-null mice there is a decrease in the density of vesicles located in the active zone and an increase in the vesicle size and in the width of synaptic clefts.	Preclinical
Vaillend et al., 2002 [21]	To determine the consequences of the increased short-term potentiation in CA1 hippocampal area, observed in *mdx* mice, on short-term depression.	Short-term depression induction is increased in CA1 area in *mdx* mice. This phenomenon may depend on altered GABA mechanisms.	Preclinical
Helleringer et al., 2018 [23]	To determine whether Dp71 loss could affect cerebellar physiology and downstream fluxes.	Dp71 deficiency selectively enhances excitatory transmission at glutamatergic synapses formed by climbing fibres on Purkinje neurons, but not at those formed by parallel fibres. Dp71 deficiency also causes impairment in synaptic plasticity and clustering of the scaffolding postsynaptic density protein PSD-95.	Preclinical
Grady et al., 2006 [24]	To explore the relationship between the dystrophin–glycoprotein complex (DGC) and dystrobrevins, as well as their role in cerebellar function in a *mdx* murine model.	DGC is required for proper maturation and function of a subset of inhibitory synapses in cerebellar Purkinje cells. Dystrobrevins are key components of the DGC. Interference with the DGC leads to behavioural abnormalities.	Preclinical
Cheng et al., 2023 [25]	To study brain connectome in DMD patients with resting-state functional magnetic resonance imaging scans.	DMD patients exhibit a significant increase in DC in the left dorsolateral prefrontal cortex (DLPFC.L) and right dorsomedial prefrontal cortex (DMPFC.R), coupled with a decrease in DC in the right cerebellum posterior lobe (CPL.R) and right precentral/postcentral gyrus.	Clinical
Rae et al., 2002 [26]	To explore the role of dystrophin in brain metabolism of *mdx* mice.	Choline-containing compound levels are increased in the cerebellum and in the hippocampus.	Preclinical
Lee et al., 2002 [28]	To determine whether regional brain glucose metabolism is altered in children with DMD and whether such dystrophin expression induces metabolic alterations.	Significant glucose hypometabolism was found in the right postcentral and middle temporal gyri, uncus, and VIIIB cerebellar lobule, as well as in the left hippocampal gyrus and cerebellar lobule. The alterations are more marked in dystrophin-rich regions.	Clinical
Golaszewski et al., 2016 [29]	To investigate motor cortex excitability in patients with Becker muscular dystrophy (BMD) through repetitive transcranial magnetic stimulation.	Patients with BMD and intellectual disability show an altered cortical short-term synaptic plasticity in glutamate-dependent excitatory circuits within the motor cortex.	Clinical
Yayla et al., 2008 [30]	To investigate the cortical excitability changes in DMD with a transcranial magnetic stimulation test battery composed of central conduction time, cortical silent period and paired TMS paradigm.	No significant alterations were found.	Clinical

### 3.2. Myotonic Dystrophy Type 1

DM1 is the most common cause of adult muscular dystrophy, caused by a pathologic expansion of a CTG trinucleotide repeat of the dystrophia myotonica protein kinase (DMPK) and transmitted through a dominant autosomal inheritance. This condition determines a multiorgan involvement with varying degrees of severity according to the dimension of the trinucleotide expansion. Although not yet completely elucidated, defects of alternative splicing and abnormal RNA accumulation in the form of nuclear ribonuclear inclusions altering the activity of RNA-binding proteins are considered the main pathogenetic mechanisms. Muscleblind-like (MBNL) proteins, such as Mbnl1 and Mbnl2, are sequestered by toxic RNA foci, while CELF family members, which promote foetal alternative splicing, are increased. In animal models, the concentration of toxic RNA foci has been demonstrated in astrocytes, cortical neurons and oligodendrocytes in multiple brain regions, including hippocampus, frontal and temporal cortex, thalamus and cerebellum [31]. CNS dysfunction has been shown to imply cognitive and behavioural abnormalities, hypersomnia, progressive memory dysfunctions and mental retardation in the congenital form, independent from the muscular involvement [32]. No disease-modifying treatment is yet available for this disorder.

#### 3.2.1. Impact on Synaptic Plasticity and Morphology

A predominant role of Mbln2 in the regulation of defective alternative splicing in CNS has been shown in Mbln2 knockout mice with a potential role in neuronal differentiation and synaptic functions [33]. Additionally, Mbln loss of function seems to cause a downregulation of astrocyte-specific GLT1 with a consequent impairment of the glutamatergic system. Therefore, an overall increase in extracellular glutamate and an insufficient compensatory increase in GABA extracellular release happens to occur in the hippocampus of transgenic *DMSXL* mice. The multiple mis-splicing events involving genes related to calcium and potassium channels, pre-synaptic and post-synaptic membrane components, and scaffolding proteins may contribute to a reduction of synaptic plasticity in DM1 [34,35]. Moreover, abnormalities in short-term plasticity have been demonstrated in the *DMSXL* hippocampus, displayed through a reduction of PPF ratios and without significant alterations of LTD and LTP compared to healthy controls [31].

#### 3.2.2. Imaging and Functional Studies

Multiple studies have shown CNS alterations in DM1 patients with morphological and functional alterations, including significant white matter disease and atrophy, reduced brain volume and cortex thickness and grey matter with association with CTG expansion size [36]. Li et al. provided evidence of increased functional connectivity of white matter as a measure of altered functional networks and possible signs of higher-order dysfunction [37]. Reductions in fractional anisotropy (FA) and increases in axial diffusivity (AD) and radial diffusivity (RD) have been described in DM1 patients, specifically in the posterior limb of the internal capsule and at the middle section of the cortico-spinal tracts (CST)—which are crucial regions for motor functions—through diffusion tensor imaging (DTI) techniques. The same authors observed a correlation between these alterations and the cortical volume reduction in sensorimotor cortex and motor performances [38].

In accordance with a further pejorative role of brain dysfunction on peripheral motor performances, FDG-PET study findings observed glucose hypometabolism in cortical areas involved in motor functions [39]. A cortical contribution to myotonia is also suggested by the detection of increased Blood oxygenation level-dependent (BOLD) signal in the supplementary motor area and the dorsal anterior cingulate cortex (dACC) in fMRI observed during grip myotonia in DM1 patients presenting myotonic phenomenon indicating an “error detection” and a “relaxation preparation” mechanism through inhibitory circuits [40].

Few works have explored the concept of altered cortical excitability in DM1 patients through neurophysiological exams. Boerio et al. studied the MEP at ADM muscle to assess a possible central component in exercise-related fatigability in DM1; although there were no significant variations in parameters before and after exercise, baseline alterations were reported with a reduction of ICF [41]. Another group employed TMS on seven DM1 patients in both M1 areas and at C7, calculating central motor conduction time (CMCT) and the peak-to-peak MEP amplitude at rest and during slight tonic contraction to characterise corticospinal excitability; the use of rapid pairs associative stimulation (rPAS) paradigm was examined to evaluate the sensory–motor plasticity. The MEPs were smaller in amplitude, with an increase in latency and abnormal morphology, and the CMCT was longer in DM1 patients in comparison with healthy controls. The finding of increased rPAS aftereffects may represent either a “use-dependent plasticity” with greater efforts for similar performances in affected patients or a phenomenon necessary to compensate for an insufficient corticospinal output. The conclusions display the possibility of exploiting the independence of the cortical sensory–motor plasticity from the myopathic component to shape functional individualised rehabilitation programs [42].

**Table 2:** Summary of the key content from articles on myotonic dystrophy.

Study	Aim of the Study	Main Findings	Type
Hernández-Hernández O et al., 2013 [31]	To enhance the understanding of the pattern of CNS involvement in DM1 mice through a global proteomics approach.	Toxic RNA foci have been demonstrated in astrocytes, cortical neurons and oligodendrocytes in hippocampus, frontal and temporal cortex, thalamus and cerebellum. RAB3A upregulation and synapsin I hyperfosforilation were linked to alteration of long-term potentiation and long-term depression.	Preclinical
Serra L et al., 2020 [32]	To analyse the implications of altered connectivity of the ventral tegmental area (VTA) in cognitive dysfunctions of adult patients with DM1 in a case–control setting through IOWA Gambling task RS-fMRI.	The study revealed a remarkable deficit in decision-making in DM1 adult patients potentially related to an increase between VTA and dopaminergic brain areas accounting for reward/punishment system and social cognition.	Clinical
Charizanis K et al., 2012 [33]	To investigate the effects of C(C)UG expression on the brain.	Mbnl2 dysregulated exons in DM determine the main pathological features through the disruption of the MBNL-2 developmental splicing program.	Preclinical
Sicot G et al., 2017 [34]	To further investigate brain cellular and molecular disruptions in the brain of DM1 mice models and adult patients.	In mice models a significant RNA toxicity in the Bergmann glia of the cerebellum together with abnormal Purkinje cell firing and fine motor incoordination. In both DM1 mice and humans, proteomics showed a downregulation of GLT1 glutamate transported secondary to MBNL1 inactivation, leading to glutamate neurotoxicity. On the contrary, the upregulation of GLT1 corrected Purkinje cell firing and motor incoordination in the DM1 mice model.	Preclinical
Potier B et al., 2022 [35]	To determine the impact of glutamate in neurotransmission in the hippocampus in a transgenic murine model and to assess changes due to the accumulation of toxic RNA in specific areas.	A reduction in the glutamate uptake in *DMSXL* mice and increased levels of glutamate and GABA currents were described. Plus, altered LTP time in DG and CA1 confirmed abnormal short-term plasticity. These synaptic alterations were described in areas where toxic RNA foci accumulation and mis-splicing of genes with relevant functions in neurotransmission were found.	Preclinical
Yoo WK et al., 2017 [36]	To investigate changes in cortical thickness and white matter structure in DM1 patients in a case–control setting using T1-weighted and diffusion tensor imaging.	In DM1 patients, compared to age-matched healthy controls, cortical thickness was significantly reduced in frontal, temporal, and occipital cortices, together with a diffuse decrease in diffusion parameters. Cortical thickness and white matter structure integrity negatively correlated with CTG repetition number in investigated areas, while no significant correlation was found between IQ scores and number of CTG repeats.	Clinical
Li J et al., 2022 [37]	To demonstrate the presence of white matter dysfunction and connectivity in DM1 patients.	Through functional MRI, DM1 patients showed increased functional connectivity in areas such as the inferior longitudinal fasciculus, prefrontal cortex, corpus callosum, superior corona radiata and deep networks, consistent with white matter dysfunction.	Clinical
Park JS et al., 2018 [38]	To investigate CNS abnormalities through MRI DTI sequences associated with motor tasks in adult DM1 patients in a case–control study.	DTI abnormalities in the corticospinal tract (CST) are associated with genetics and motor features (i.e., clinical myotonia), alongside a positive correlation between cortical grey matter volume and DTI parameters in the CST.	Clinical
Peric S et al., 2017 [39]	To assess reduction in brain metabolism through PET-FDG in DM1 and DM2 patients and its association with neuropsychological deficits.	The reduction of metabolism in areas involved in executive, visuospatial, and naming dysfunction well correlates with neuropsychological findings in DM1 suggesting a possible role of the FDG-PET as a cognitive biomarker.	Clinical
Toth A et al., 2015 [40]	To investigate cortical abnormalities during grip myotonia in adult DM1 patients in a case–control study through fMRI.	Myotonia correlated with increased BOLD signal in supplementary motor area and dorsal anterior cingulate cortex.	Clinical
Boërio D et al., 2012 [41]	To assess the central and peripheral components of exercise-related fatigability in patients with DM1.	While DM1 patients baseline peripheral and central excitability was altered compared to controls, no significant changes in the excitability parameters were found after exercise.	Clinical
Portaro S et al., 2017 [42]	To study central motor conduction, sensory–motor plasticity and excitability in brain cortex of DM1 adult patients through TMS in a case–control study.	An increased central motor conduction time and disrupted neural plasticity in the sensory–motor system was found in DM1 patients, despite no changes in cortical excitability.	Clinical

### 3.3. Other Muscle Diseases

#### 3.3.1. Systemic Autoimmune Myopathies

Systemic autoimmune myopathies (SAMs), or idiopathic inflammatory myopathies, are autoimmune diseases characterised by symmetrical and predominantly proximal muscle weakness. Considering clinical, laboratory, and histopathological features, SAMs can be classified in dermatomyositis (DM), clinically amyopathic dermatomyositis (CADM), polymyositis (PM), antisynthetase syndrome (ASSD), immune-mediated necrotising myopathies (IMNM), and inclusion body myositis (IBM). While describing the specific physiopathological mechanisms is beyond the scope of this review, there are features involving peripheral and central fatigue that are common to different autoimmune diseases [43]; moreover, tDCS has already been proven efficacious in systemic rheumatologic diseases on pain, fatigue and weakness symptoms. In 2021, de Sousa et al. evaluated a tDCS protocol of stimulation of M1 with a randomised double-blind trial. A total of 20 patients with DM or PM were recruited and divided into an active group (3 daily sessions of 30 min of anodal stimulation on C1 or C2, contralateral to the dominant limb, and cathode on Fp1-Fp2) and a sham group (stimulation only lasted 30 s). Investigators found some improvement in strength measured through the 30 s Time Sit to Stand test (TSST) in both groups, which lasted at 3 and 8 weeks after the tDCS sessions only in the active stimulation group; however, strength measured through isokinetic tests and QoL outcomes did not differ significantly among the two groups [44]. The same group conducted a further placebo-controlled randomised trial on 31 patients with DM, CADM or IMNM with a different protocol (10 daily sessions of 30 min primary motor cortex stimulation followed by 30 min of treadmill walking). The active group reported an improvement in Functional Index-3 in cervical flexion and hip flexion exercises, with some effect also on pain intensity; again, QoL did not significantly improve [45].

#### 3.3.2. Spinal Muscular Atrophy (SMA)

SMA is an autosomal recessive disease characterised by proximal, progressive, symmetric weakness and muscle atrophy. The disease is caused by mutations or deletions in the SMN1 gene, which results in the absence of the SMN protein with loss of spinal motor neurons. Increasing evidence is linking SMN deficit to defects in synaptic maintenance, both in CNS and neuromuscular junction (NMJ) [46,47].

*Smn 2^B/−^* mouse models show disorganisation and reduction of muscular endplates, which is proportional to the functional involvement of the examined muscle. Moreover, after traumatic nerve injury, while the axon growth rate was similar in controls and *Smn 2^B/−^* mice, NMJ remodelling was impaired in the latter. A defective re-organisation of acetylcholine receptors was observed only in nerve-directed post-synaptic remodelling, while acetylcholine receptor clustering was normal in nerve-independent remodelling. Authors suggested that these defects may be due to alterations in nerve-specific signalling or in the communication between muscle and nerve [48].

In humans, an fMRI study identified altered nodal properties involving the cortical–limbic–cerebellum circuitry; nodal properties, in particular, the nodal degree of the right cerebellar lobule VII and the nodal degree of the right amygdala, showed a correlation to disease duration and functional scores. These findings suggest that compensatory mechanisms increase the FC in non-motor areas to maintain normal cognitive functions [49].

Altogether, it seems plausible that plasticity mechanisms are altered both in peripheral and central synapses, leading to speculations on possible future therapeutic approaches. 

#### 3.3.3. Preclinical Findings in Other Myopathies

Other genes connected to muscle disorders have been linked to changes in synaptic organisation and plasticity in animal models. 

Dynamin-2 (Dyn2), a large GTPase that regulates the polymerisation of actin, is known as a cause of autosomal dominant centronuclear myopathy (CNM). Dyn2 mutations alter actin-dependant processes, including exocytosis, endocytosis and membrane remodelling not only in skeletal muscle but also in CNS: mice carrying the p.R465W mutation, the most common disease-causing variant, showed a reduced dendritic branching, a shorter total length of dendrites and fewer dendritic intersection in primary hippocampal neurons. Moreover, synaptic plasticity was altered as well in the same population of neurons: after induction with glycine and NMDA, respectively, LTD and LTP were impaired in Dyn2-mutated neurons; considering that this approach bypasses synaptic mechanisms, these changes in synaptic plasticity appear to depend on postsynaptic mechanisms. Indeed, Dyn2 is known to modulate the membrane insertion, removal and recycling of the α-amino-3-hydroxy-5-methyl-4-isoxazole propionic acid (AMPA) receptors at the postsynaptic level. Considering the importance of LTD and LTP on learning and memorisation, it is not surprising that Dyn2-mutated mice showed reduced recognition memory, confirming even in animal models the reports of learning impairment in patients with CNM [50]. Alterations in LTD and LTP have also been observed in mouse models of laminin α2-related congenital muscular dystrophy (LAMA-2) and in α-dystroglycan (α-DG) muscular dystrophy models. Laminin and dystroglycan are proteins functionally related to dystrophin: α-DG docks with β-dystroglycan (β-DG) to form the core of the dystrophin-associated glycoprotein complex (DGC), which links proteins in the extracellular matrix, such as agrin, pikachurin, and perlecan, to dystrophin. In CNS synapses, α-DG seems to play a crucial role in regulating the clustering of GABA_A_ receptors in the hippocampus, similar to the regulation of acetylcholine receptor clustering that it exerts in NMJ [51,52]. The reduction of inhibiting synapses caused by α-DG dysfunction may play a role in the enhancement of LTP previously described in *mdx* mice and in the incidence of epilepsy observed in DGC-related muscular dystrophy (including LAMA-2) and in DMD [53]. On the other hand, laminin α2 binds DG, helping the anchoring of the complex [54]. Laminin-deficient mice showed an impairment of LTD in cerebellar Purkinje cells, leading to a deficit in motor learning. Of interest, both laminin and DG play a crucial role in the developmental phase of the brain by guiding axon growth, while in the adult, their expression is more relevant in specific areas, namely the hippocampus, the limbic system and the cerebellum. The interconnection among DG, laminin and dystrophin and their role in regulating neuronal plasticity mechanisms may explain the learning and motor deficit observed in muscular dystrophies related to a mutation of these proteins, further elucidating the extent of such involvement may help to tailor a specific treatment for cognitive symptoms even through the means of neurostimulation techniques [55].

#### 3.3.4. Neurophysiological Findings in Other Myopathies

In other myopathies, neurophysiological studies on humans have given contrasting findings. In facioscapulohumeral muscular dystrophy (FSHD), an autosomal dominant myopathy whose pathogenetic mechanisms are not fully cleared yet, central involvement is described, with epilepsy and cognitive impairment. In FSHD, TMS studies showed a significant motor cortex ICI reduction compared to healthy controls. While these findings were only partially replicated in other myopathies, resting motor threshold (RMT) appears to be higher in amplitude in patients with myopathies compared to controls, regardless of the specific diagnosis [56,57]. ICI is thought to depend on mechanisms related to GABA_A_ receptors, while RMT is a more complex measure that reflects the excitability of cortex axons and synapses [58]. While RMT changes may be attributed to mechanisms of synaptic plasticity secondary to muscle denervation common to all myopathies, ICI impairment may reveal a GABAergic dysfunction specific to only some of them, FSHD above all.

Changes in cortical excitability have been investigated only preliminarily in inflammatory myopathies: two patients with polymyositis were included in the aforementioned study by Di Lazzaro et al., showing an increase in RMT compared to controls, similar to inherited myopathies [56]. However, Lin et al. analysed MEPs, CMT, cortical silent period and ICI in eight patients with colchicine myopathy without significant differences from controls [59].

While the sample sizes of these studies were too small to draw definite conclusions, the lack of cortical excitability alterations in inflammatory myopathies may be attributed to specific molecular mechanisms that are peculiar to certain genetic diseases or to the shorter disease duration time; in fact, ICI impairment in FSHD was significantly related to age, while in the study by Lin et al. on colchicine myopathy only 2/8 patients had a disease duration > 6 months [56,59].

There are myopathies with muscle stiffness and cramps, such as myotonic dystrophy type 2, Brody disease (caused by a mutation of the sarcoplasmic/endoplasmic reticulum Ca^2+^ ATPase type 1 [SERCA1], the main protein involved in muscle relaxation), McArdle disease (glycogenosis type 5) and nemaline myopathy type 6, in which TMS-induced muscle relaxation is slower than healthy controls or patients with myopathies without muscle stiffness. This measure proved to be highly reliable in the distinction between myopathies with and without myotonia; further testing on bigger samples may lead to the standardisation of the technique for clinical practice, thus introducing an additional test that may provide useful information and is faster and less invasive and expensive than muscle biopsy or genetic testing [60].

**Table 3:** Summary of the key content from articles on other acquired and hereditary neuromuscular conditions.

Study	Aim of the Study	Main Findings	Type
de Sousa et al., 2022 [44]	To assess the safety and efficacy of transcranial direct current stimulation (tDCS) in patients with idiopathic inflammatory myopathy.	Slight improvement in strength in both active and control group (which lasted at 8 weeks only in the active group), emotional aspects significantly improved only in the active group.	Clinical
Missé et al., 2024 [45]	To assess the effectiveness of transcranial direct current stimulation (tDCS) for pain, fatigue and quality of life in patients with idiopathic inflammatory myopathy.	Pain and fatigue improved in the active group.	Clinical
Wishart et al., 2010 [47]	To investigate the role of SMN protein in brain development and maintenance in a mouse model.	Reduced levels of SMN lead to impaired perinatal brain development in areas normally associated with higher SMN levels, such as the hippocampus.	Preclinical
Murray et al., 2013 [48]	To investigate the role of SMN protein in neuromuscular junction (NMJ) in a mouse model.	*Smn 2^B/−^* mouse models show disorganisation and reduction of muscular endplates which is proportional to the functional involvement of the examined muscle. Nerve-directed re-organisation of acetylcholine receptors is altered.	Preclinical
Li et al., 2024 [49]	To explore the brain neural network mechanisms in SMA patients with morphological brain network analysis.	SMA patients exhibit altered nodal properties involving the cortical–limbic–cerebellum circuitry.	Clinical
Arriagada-Diaz et al., 2023 [50]	To study the influence on central nervous system functioning of a dynamin-2 centronuclear myopathy-causing mutation.	Hippocampal neurons exhibited reduced dendritic arborisation and lower spine density. Excitatory synaptic transmission was altered in hippocampal neurons.	Preclinical
Pribiag et al., 2014 [53]	To study the influence on central nervous system of α-dystroglycan (α-DG) in a mouse model.	α-DG dysfunction causes a reduction of inhibiting synapses in hippocampal inhibitory neurons.	Preclinical
Moore et al., 2002 [55]	To investigate the role of dystroglycan deletion on brain function in a mouse model.	Mutant mice exhibit severely impairment of hippocampal long-term potentiation.	Preclinical
Lazzaro et al., 2004 [56]	To investigate functional brain changes in adults with FSHD through TMS in a case–control study.	A reduced cortical inhibition was found in FSHD patients when compared to controls and other myopathic patients.	Clinical
Liepert et al., 2004 [57]	To study alterations of motor neuronal excitability in patients with structural genetic myopathies through TMS and electrical nerve stimulation in a case–control study.	A reduction of cortical inhibition together with increased in alpha-motor neuron excitability and motor-evoked potentials during contraction were detected in myopathic patients.	Clinical
Lin et al., 2010 [59]	To study central compensation in patients with colchicine myopathy experiencing fatigue trough TMS in a case–control setting.	No remarkable changes in cortical excitability were evidenced in a cohort of colchicine myopathy patients.	Clinical
Molenaar et al., 2023 [60]	To investigate muscle relaxation patterns in myopathies characterised by stiffness and cramps/myalgias through TMS in a case–symptomatic control study.	In men with Brody disease, nemaline myopathy type 6 (NEM6) and myotonic dystrophy type 2 a lower peak relaxation rate was identified. On the counter side, the same results were obtained in women with NEM6 and McArdle disease. TMS yielded different results distinguishing symptomatic controls from myopathic patients.	Clinical

## 4. Therapeutic Perspectives

The employment of functional and NIBS techniques to improve motor performances acting on synaptic plasticity is progressively expanding. Some of the observed alterations, particularly in Dp71-deficient neurons, may suggest a maladaptive response. However, changes in cortical excitability and functional connectivity, notably in DM1 and SMA, point to potential plastic adaptations. Preclinical evidence indicates that these alterations may be primarily driven by disease-specific molecular deficits, rather than secondary to muscle weakness. In this context, such deficits lead to synaptic dysfunction, which is compensated by increased CNS excitability in other functional circuits. Nonetheless, to further confirm this hypothesis and elucidate the possible role of secondary changes in brain plasticity, further clinical studies with larger cohorts and the inclusion of subjects with preclinical or subclinical deficits are required. These compensatory mechanisms are thought to help preserve motor and cognitive functions. A more comprehensive understanding of these adaptive modifications could guide the development of targeted therapies aimed at enhancing and sustaining brain plasticity. Here, we will examine current applications and future prospects for NMDs, as well as the effects that current treatments may induce on cortical plasticity.

### 4.1. Dystrophinopathies

Given the significant impact of dystrophin deficiency on CNS function, several therapeutic strategies have been explored. One therapeutic avenue involves the use of rTMS, which can modulate cortical excitability by inducing short-term increases in synaptic efficiency [29]. This technique may hold the potential to compensate for dystrophin-dependent alterations in synaptic plasticity in DMD patients, particularly in regions like the motor cortex.

From a pharmacological perspective, in models with Dp71 deficiency, the restoration of dystrophin-like proteins through exon skipping has been shown to normalise synaptic plasticity in the hippocampus. For example, U7 small nuclear RNAs engineered to encode antisense sequences have been used to induce skipping of the mutated exon 23, resulting in the rescue of a functional dystrophin-like product in both muscle and nervous tissue. In the brain, this rescue was accompanied by the restoration of normal hippocampal GABA_A_ receptor clustering and the reversal of the abnormally enhanced LTP phenotype at CA3-CA1 [61].

Additionally, modulation of the Wnt signalling pathway has shown potential as a therapeutic strategy. The Wnt pathway plays a crucial role in neuronal development and synaptic maintenance, and its modulation has been demonstrated to improve central inhibitory synaptic transmission in a mouse model of DMD. Enhancing GABAergic efficacy through Wnt signalling could increase the number of inhibitory synapses and GABA_A_ receptors, thereby restoring the excitation–inhibition balance that is disrupted in Dp71-deficient brains [62].

### 4.2. Myotonic Dystrophy Type 1 (DM1)

In DM1, some small-sized pilot studies have been carried out to determine a possible benefit to motor performance. A fortnight protocol with a twice-a-day home session of neuromuscular electrical stimulation was adopted and achieved evidence of an improvement in motor strength and functional motor performances in a cohort of five DM1 patients. The positive results—although transient and selective—suggested to include this technique in the early rehabilitation of DM1 patients [63]. Another pilot study was conducted to assess the utility of a wearable multifocal TMS device called a Transcranial Rotating Permanent Magnet Stimulator able to deliver simultaneously or sequentially focal low-intensity magnetic stimuli (the peak amplitude is 7% of maximal TMS output) or sham signals. Six adult DM1 patients underwent a 15-day trial with one-sided stimulation of the M1 cortex and sham signal on the other side. The results did not show any significant improvement of the MRC scale or the dynamometer score at the hang grip test, but an increase in the compound motor action potential amplitude on the stimulated side compared to the pre-treatment data and to the not-treated side. In consideration of the feasibility of the machine and the good tolerance of the instrument, it was proposed to consider longer treatment intervals and larger cohorts to attest to the safety and efficacy of chronic therapy [64]. Even if the use of robotic devices in neuromuscular conditions is not common yet, a case report describes the use of a robotic exoskeleton in a DM1 patient rehabilitation program with improvement in functional motor performances, gait analysis and modulation of functional connectivity with strengthening of prefrontal connections [65].

### 4.3. Other Myopathies

Interventional protocols on SAMs have already been discussed in detail in Section 3.3.1 [44,45]. Despite the solid theoretical bases, these studies found only modest effects of tDCS on strength and fatigue. This could be due to some limitations: the small sample size, the exclusive inclusion of patients in remission or minimal disease activity, which therefore have smaller margins of improvement, and the reduced number of sessions. The little to no effect on pain recorded compared to trials conducted on Sjögren’s syndrome [66] or fibromyalgia [67], as the authors noted, might be due to the different targets of stimulation. Moreover, isokinetic strength improvement after tDCS is debated either in normal subjects [68]; therefore, other outcome measures may be needed to better understand the impact on strength and fatigue, such as grip force, walking velocity and walking capacity, as seen in tDCS protocols conducted on stroke and multiple sclerosis [68,69].

## 5. Study Limitations

This study has some limitations. First, many of the studies reviewed focused on preclinical models, which require further validation through clinical trials to confirm the relevance of their findings in human populations. Additionally, the clinical studies often had small sample sizes, lacked comprehensive longitudinal assessments, or were conducted over short timeframes. The heterogeneity in the number of studies addressing each disease further complicated the ability to draw definitive conclusions. Lastly, in interventional studies, beyond issues of sample size and follow-up, outcome measures and neurostimulation protocols were often too varied or inconsistent with previous evidence, potentially affecting the assessment of the techniques’ efficacy.

## 6. Conclusions

Emerging evidence suggests that synaptic plasticity mechanisms may be involved in the pathogenesis of various muscular diseases, not only in relation to central and cognitive symptoms but also in terms of strength and fatigue. While some features of these mechanisms have been thoroughly investigated, many critical questions remain unanswered. These include the relationship of plasticity in specific genetic forms, the extent of its involvement in inflammatory myopathies, and the potential for targeting these mechanisms through pharmacological, neurostimulation or rehabilitation interventions.

In the era of emerging disease-modifying therapies for genetic NMDs, a deeper knowledge of the mechanisms of CNS involvement could lead to the development of more tailored therapies. Currently, the current literature shows a modest interest in CNS therapeutic approaches, as well as outcomes considering brain plasticity.

Ideally, future clinical trials will explore the potential of these treatments to impact CNS function, using measures capable of assessing the dynamics of brain plasticity.

The rarity of these diseases, combined with the heterogeneity of their phenotypic presentations, presents considerable challenges for researchers in this field. Recent studies demonstrating the therapeutic potential of targeting synaptic plasticity in muscular diseases should motivate increasing focus and investment in this area of research.
